# Acupuncture and related therapies for hyperlipidemia: A protocol for systematic review and network meta-analysis

**DOI:** 10.1097/MD.0000000000023548

**Published:** 2020-12-04

**Authors:** Xue-Song Wang, Jia-Jia Li, Yue-Shen Wang, Chao-Chao Yu, Chuan He, Zhong-Sheng Huang, Miao Wu, Li-Hong Kong

**Affiliations:** a Hubei University of Chinese Medicine; b Preventive Treatment of Acupuncture and Moxibustion of Hubei Provincial Collaborative Innovation Center, Wuhan, Hubei; c Shenzhen Traditional Chinese Medicine Hospital, Shenzhen, Guangzhou; d Hubei Provincial Hospital of Traditional Chinese Medicine; e Hubei Province Academy of Traditional Chinese Medicine, Wuhan, Hubei, China.

**Keywords:** acupuncture, high density lipoprotein cholesterol (HDL-C), hyperlipidemia, low density lipoprotein cholesterol (LDL-C), Network Meta-Analysis (NMA), total cholesterol (TC), triacylglycerol (TG)

## Abstract

**Objective:**

To compare and rank the clinical effects of different acupuncture and acupuncture-related therapies on patients with hyperlipidemia.

**Methods:**

We used Network Meta-Analysis (NMA) to evaluate the direct and indirect evidence from relevant studies. Three English and 4 Chinese databases were searched to collect randomized controlled trials (RCT) of acupuncture and related therapies in the treatment of hyperlipidemia. The data were analyzed using Stata15.0 and WinBUGS1.4.3 software after 2 researchers independently screened the literature, extracted the data, and assessed the risk of bias in the included studies.

**Results:**

Based on the current evidence, we comprehensively compare the pros and cons of various acupuncture-related therapies, rank the efficacy of various acupuncture-related therapies compared with statins in the treatment of hyperlipidemia, and summarize the best acupuncture intervention methods or combinations.

**Conclusion:**

This study will provide new evidence for the safety and effectiveness of acupuncture-related therapies in the treatment of hyperlipidemia, and may be helpful for clinicians, hyperlipidemia patients, and clinical guideline makers to choose the optimal combination of acupuncture for the treatment of hyperlipidemia.

**Registration number:**

INPLASY2020100100

## 1 Introduction

Hyperlipidemia is a disorder of lipid metabolism characterized by an increase in triacylglycerol (TG), total cholesterol (TC), low density lipoprotein cholesterol (LDL-C), and a decrease in high density lipoprotein cholesterol (HDL-C) in the peripheral blood, and is associated with hypertension, diabetes, and obesity as major vascular risk factors.^[1,2]^ The incidence of hyperlipidemia is increasing year by year with the improvement of people living standards and changes in lifestyle. According to the 2016 guidelines for the prevention and treatment of dyslipidemia in adults in China, the prevalence of dyslipidemia among adults in China is as high as 40.40%, a substantial increase from 2002,^[3]^ and the proportion of children and adolescents with hypercholesterolemia in China is also significantly higher.^[4]^ Suggesting that the burden of dyslipidemia and related diseases in China will continue to increase in the future.^[5]^ Hyperlipidemia is closely related to the occurrence of coronary heart disease and stroke, and may induce Alzheimer disease (AD), vascular dementia (VD), and Parkinson disease (PD), etc.^[6,7]^ At present, statins, beta-blockers, and other lipid-lowering drugs are mainly used in clinical practice, which have some efficacy, but may cause adverse effects such as liver damage, rhabdomyolysis, neoplasia, and diabetes.^[8]^ Due to the complex etiology of hyperlipidemia, it is closely related to environment and genetics, but its mechanism has not been fully elucidated. Acupuncture, as the most representative non-pharmacological therapy in traditional Chinese medicine, without the side effects and clinical contraindications of Western medicine, is a treatment method with potential for development. In recent years, more and more studies have been conducted on the use of acupuncture to treat patients with hyperlipidemia, and previous meta-analyses had found that for patients with hyperlipidemia, acupuncture,^[9–11]^ auricular acupuncture,^[12]^ and herbal medicine^[13]^ may be more advantageous and safer than Western medicine.^[9,10]^ However, due to the wide variety of acupuncture and the different focus on efficacy, there is still a lack of direct comparative studies between different acupuncture-related therapies. In this study, the Network Meta-Analysis (NMA) method was used to evaluate the effects of various acupuncture-related therapies for patients with Hyperlipidemia, expected to provide evidence-based medicine evidence for selecting the best combination of options.

## 2 Methods

It will be reported following the Preferred Reporting Items For Systematic Reviews And Meta-Analyses for Network Meta-Analysis Checklist (PRISMA-NMA).^[14]^ This study has been registered with INPLASY, and registration number was INPLASY2020100100.

### 2.1 Search strategies

Our literature search was performed from database establishment until April 1st, 2020, including 3 English databases: PubMed, EMBASE, Cochrane Library, and 4 Chinese databases: the China Biology Medicine (CBM), the China National Knowledge Infrastructure (CNKI), Wanfang Data, the Chinese Scientific Journal Database (VIP). The search was conducted using a combination of medical subject headings (MeSH) terms and free words. In addition, the references included in the medical literature were retrospectively supplemented to obtain associated references.

### 2.2 Inclusion criteria

**Figure 1:**
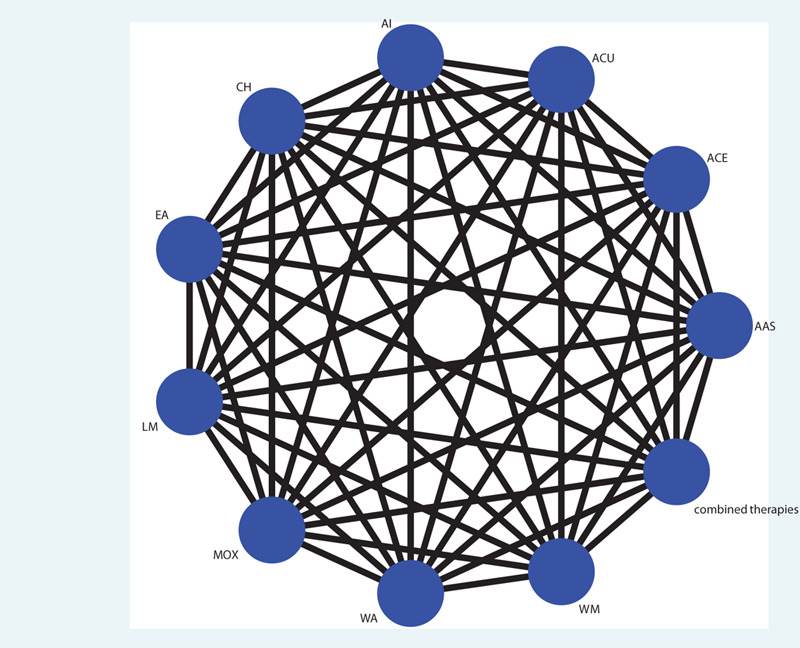
Network plot of possible direct comparisons.

The published randomized controlled trials (RCT) of acupuncture-related therapies for the treatment of primary hyperlipidemia, regardless of age and gender. Clear diagnostic criteria were required to confirm the diagnosis of primary hyperlipidemia. Interventions in the treatment group were various types of acupuncture-related therapies, including simple acupuncture, electroacupuncture, warm acupuncture, auricular acupuncture, acupuncture point injections, acupoint embedding, or a combination of acupuncture and drugs; the control group is statin lipid-lowering western medicine, or placebo, or comparison between various acupuncture-related therapies. Comparisons investigated are given in Figure 1. The results of report are required to include at least one of the following outcome indicators: TC, TG, LDL-C, HDL-C. The language of the publication is limited to Chinese or English.

### 2.3 Exclusion criteria

1. Research on self-controlled or other non-randomized controlled trials;
2. Research with unclear diagnostic criteria;
3. Research on subjects belonging to secondary hyperlipidemia (such as obesity, diabetes, etc.);
4. Pre-clinical studies, systematic reviews, case reports, and meta-analysis, etc.;
5. The report did not have clear original data, and contacting the author was unsuccessful;
6. There was no acupuncture-related therapy or other forms of acupuncture (such as transcutaneous electrical nerve stimulation or transcranial magnetic stimulation, etc.);
7. Repeated research or research report results are the same.

### 2.4 Literature screening and data extraction

Two researchers, ZH and CH, independently conducted the literature screen, data extraction, and cross-check. In any case of disagreement, they would discuss to reach a consensus or a third researcher, CY would assist in the final determination. A unified data extraction table was used for data extraction, which included: including general information of the included literature:

1. the name of the first author, journal name, publication year, etc.;
2. baseline data of the subjects included in the literature: grouping, the sample size of each group, and Subjects age, etc.;
3. intervention methods: type of intervention, treatment frequency and treatment period, etc.;
4. risk bias related factors: random method, allocation hiding, blinding, etc.;
5. outcome indicators before and after treatment data.

### 2.5 Risk assessment of bias in inclusion studies

Our 2 researchers, JL and YW, evaluated the included studies in accordance with the bias risk assessment tool recommended on Cochrane Handbook 5.1.^[15,16]^ However, acupuncture-related therapies (such as simple acupuncture, electroacupuncture, warm acupuncture, auricular acupuncture, etc.) were non-pharmacological therapies, therefore some participants and researchers involved in these studies were not able to be blinded. So we required be blinded for the outcome assessment.

### 2.6 Statistical analysis

Statistical analysis was performed using Stata 15.0 and WinBUGS 1.4.3 software.^[17–19]^ TC, TG, LDL-C, HDL-C are numerical variables, and the difference before and after treatment is used as the effect size. In some trials, the change between baseline and after treatment failed to show, and the missing data were estimated using the following formula:^[16]^

(1) $${\bar{\text{X}}}_{change}={\bar{\text{X}}}_{post-treatment}-{\bar{\text{X}}}_{baseline}$$

(2) $$\text{S}{\text{D}}_{\text{change}}=\sqrt{{\left(\text{S}{\text{D}}_{\text{baseline}}\right)}^{2}+{\left(\text{S}{\text{D}}_{\text{post-treatment}}\right)}^{2}-2\times r\times \text{S}{\text{D}}_{\text{baseline}}\times \text{S}{\text{D}}_{\text{post-treatment}}}$$

First, Stata15.0 was used to draw an NMA evidence relationship diagram;^[20]^ then WinBugs1.43 was run to set the number of iterations to 50,000 for NMA; 95% confidence interval (95% CI) of inconsistency factors (IF) was used to judge the consistency of the closed-loop. If the IF with 95% CI contains 0, it means that the direct and indirect evidence is consistent, otherwise, it means that there is a higher possibility of inconsistency.^[21]^ Second, the Stata 15.0 program was applied to create funnel plots to determine whether there was evidence of small sample effects in the included studies.^[22]^ Finally, the surface under the cumulative ranking curve (SUCRA) was generated using Stata 15.0 to show the SUCRA scores for all interventions, with higher SUCRA scores implying higher treatment class.^[23]^

## 3 Discussion

With the improvement of people living standards and changes in lifestyles, hyperlipidemia has become an important public health problem, and its incidence has increased significantly in all age groups. Dyslipidemia is an important vascular risk factor, which is closely related to atherosclerosis and stroke. Studies have shown that in Asian populations, for every 1 mmol/L increase in peripheral blood cholesterol, the incidence of cardiovascular disease will increase by 35%, and the incidence of stroke may increase by 25%.^[24]^ Lowering cholesterol is effective in reducing the incidence and mortality of coronary heart disease, and it plays an important role in the prevention and treatment of coronary heart disease. Currently, the drugs used to treat hyperlipidemia (e.g., statins and beta-blockers) have different degrees of adverse effects, and the treatment of lipid-lowering drugs needs to be individualized and tailored, and adverse effects need to be monitored during treatment. It is necessary to pay attention to the adverse effects and regularly test the liver function. And serum creatine kinase, liver function damage, and rhabdomyolysis caused by clinical drug treatment cannot be ignored. Therefore, it is particularly important to find safe and effective treatments for reducing blood lipids.

Acupuncture, as a characteristic therapy of traditional Chinese medicine, has a good effect for many diseases in the clinic, which can be used as a safe and effective complementary alternative therapy for hyperlipidemia, considering the safe and side effect-free characteristics of acupuncture operation. It is necessary to scientifically evaluate the curative effect of acupuncture on hyperlipidemia in order to evaluate the curative effect-cost relationship of acupuncture to select the best acupuncture Methods or the best combination of treatments can better reduce the economic burden of patients. This study uses MNA to make up for the lack of direct data. Indirect data is used to compare the efficacy of different acupuncture-related therapies in the treatment of hyperlipidemia, which provides some evidence-based medical evidence for the clinical efficacy of acupuncture-related therapies for hyperlipidemia.

Based on the current evidence, we comprehensively compare the pros and cons of various acupuncture-related therapies, rank the efficacy of various acupuncture-related therapies compared with statins in the treatment of hyperlipidemia, and summarize the best acupuncture intervention methods or combinations. Reliable evidence will be obtained for acupuncture-related therapies for the treatment of hyperlipidemia. This study will provide new evidence for the safety and effectiveness of various acupuncture-related therapies as a complementary alternative therapy for hyperlipidemia, it may be helpful for clinicians, hyperlipidemia patients, and clinical guideline makers to choose the optimal combination of acupuncture t for the treatment of hyperlipidemia.

## Author contributions

**Data curation:** Xue-Song Wang, Jia-Jia Li.

**Formal analysis:** Chuan He, Zhong-Sheng Huang.

**Funding acquisition:** Li-Hong Kong.

**Methodology:** Xue-Song Wang, Yue-Shen Wang.

**Project administration:** Li-Hong Kong, Miao Wu.

**Resources:** Jia-Jia Li, Yue-Shen Wang.

**Software:** Xue-Song Wang.

**Supervision:** Miao Wu, Li-Hong Kong.

**Validation:** Chao-Chao Yu.

**Writing – original draft:** Xue-Song Wang, Jia-Jia Li, Yue-Shen Wang.

**Writing – review & editing:** Chao-Chao Yu; Miao Wu, Li-Hong Kong.
